# Sophisticated yet Convenient Information Encryption/Decryption Based on Synergistically Time‐/Temperature‐Resolved Photonic Inks

**DOI:** 10.1002/advs.202206290

**Published:** 2022-12-11

**Authors:** Dong Li, Jia‐Min Wu, Zheng‐Hong Liang, Lin‐Yue Li, Xiu Dong, Si‐Kai Chen, Teng Fu, Xiu‐Li Wang, Yu‐Zhong Wang, Fei Song

**Affiliations:** ^1^ The Collaborative Innovation Center for Eco‐Friendly and Fire‐Safety Polymeric Materials (MoE) National Engineering Laboratory of Eco‐Friendly Polymeric Materials (Sichuan) State Key Laboratory of Polymer Materials Engineering College of Chemistry Sichuan University Chengdu 610064 P. R. China

**Keywords:** anticounterfeiting, information encryption, photonic ink, temperature‐resolved decryption, time‐resolved decryption

## Abstract

Exploring high‐safety but convenient encryption and decryption technologies to combat threats of information leakage is urgently needed but remains a great challenge. Here, a synergistically time‐ and temperature‐resolved information coding/decoding solution based on functional photonic inks is demonstrated. Encrypted messages can be stored into multiple channels with dynamic‐color patterns, and information decryption is only enabled at appointed temperature and time points. Notably, the ink can be easily processed into quick‐response codes and multipixel plates. With high transparency and responsive color variations controlled by ink compositions and ambient temperatures, advanced 3D stacking multichannel coding and Morse coding techniques can be applied for multi‐information storage, complex anticounterfeiting, and information interference. This study paves an avenue for the design and development of dynamic photonic inks and complex encryption technologies for high‐end anticounterfeiting applications.

## Graphical abstract

Here, a synergistically time‐/temperature‐resolved information coding/decoding solution based on a photonic anticounterfeiting ink is demonstrated. The ink can be easily processed into different patterns, which enable the storage of messages into multiple channels as well as the information decryption at appointed temperature and time points, providing inspirations for advanced information‐encryption and anticounterfeiting technologies.

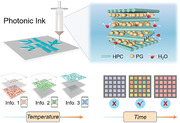

## 1 Introduction

Nowadays, information security has imposed a tremendous impact on human living, social stability, and even national security, causing urgent requirements of anticounterfeiting materials and advanced encryption/decryption technologies.^[^ ^1^, ^2^, ^3^, ^4^, ^5^, ^6^ ^]^ To this end, anticounterfeiting materials, including watermarks,^[^ ^7^, ^8^, ^9^ ^]^ photonic crystals,^[^ ^10^, ^11^, ^12^, ^13^, ^14^, ^15^, ^16^, ^17^, ^18^, ^19^ ^]^ perovskite nanocrystals,^[^ ^20^, ^21^, ^22^ ^]^ and luminescent patterns,^[^ ^23^, ^24^, ^25^, ^26^, ^27^, ^28^ ^]^ have been created to combat threats caused by fake information and information leakage. Versatile stimuli, including pH, light, and chemical and mechanical signals, have been explored as decryption keys to induce changes in appearance and properties of such materials that are visible to naked eyes or verified with special instruments.^[^ ^29^, ^30^, ^31^, ^32^, ^33^, ^34^, ^35^, ^36^, ^37^ ^]^ However, suffering simple single‐channel decryption, traditional anticounterfeiting materials, and technologies are easily duplicated. Thus, the following two main strategies have been proposed: one is the incorporation of multiple anticounterfeiting materials into a product,^[^ ^38^, ^39^, ^40^, ^41^, ^42^ ^]^ and the other is using multiple stimuli as a collaborative key for information decryption.^[^ ^43^, ^44^, ^45^, ^46^, ^47^, ^48^ ^]^ Nevertheless, integration of different categorical anticounterfeiting materials generally requires complicated processing and is of high time‐consumption, while complex decoding operations may bring critical requirements for decryption devices. Hence, encryption technologies featuring high‐security materials and facile decryption manipulations are urgently desired but remain challenging.

**Scheme 1:**
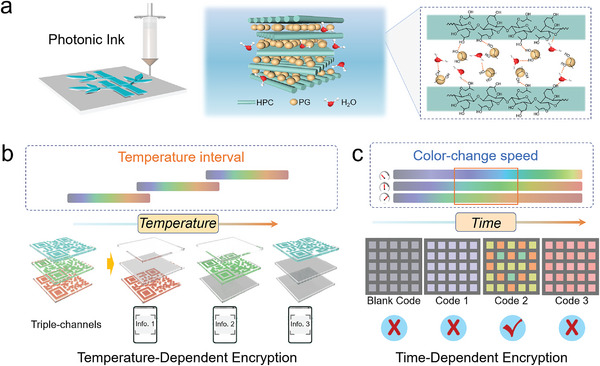
HPC/PG mesophase materials for advanced information encryption. a) Schematic illustration for the preparation of cholesteric HPC/PG mesophase. b) Temperature‐resolved and c) time‐resolved information encryption and decryption.

In this work, we demonstrate a new photonic anticounterfeiting ink that has color variations discerned by naked eyes using a simple and convenient binary decryption key (temperature and time points). Structural colors of such ink made from hydroxypropyl cellulose (HPC)/propylene glycol (PG) mesophases can be modulated from colorless and transparent to the whole visible‐light region by the composition and environmental temperature. More importantly, the ink can be easily processed into complex patterns, quick‐response (QR) codes, and multipixel plates (**Scheme** **1**). By using the ink, encrypted information can be meticulously programmed at certain temperatures (by the HPC phase) and time points (by the PG phase). Advanced encryption technologies, including multichannel and Morse coding (Scheme 1b,c), can be applied for improving information‐storage security and decoding complexity. This time‐ and temperature‐resolved encryption and decryption strategy, with features of sophisticated yet convenient information coding and decoding, offers a new way for high‐level anticounterfeiting applications.

## 2 Results and Discussion

**Figure 1:**
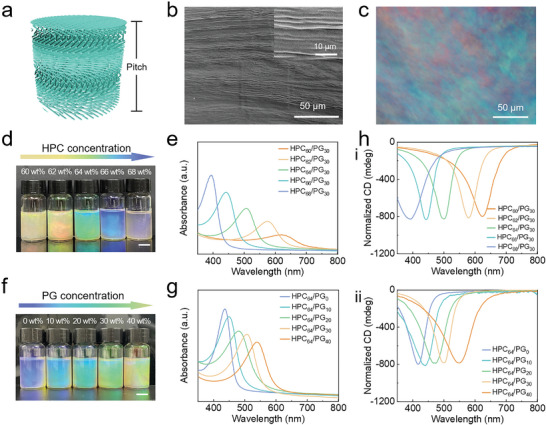
Structures and coloration of HPC/PG mesophases. a) Schematic illustration of the cholesteric structure of HPC/PG mesophases. b) A cross‐sectional SEM image and c) a POM image of a typical HPC/PG mesophase. Optical images and UV‐vis extinction spectra of HPC/PG mesophases with different d,e) HPC and f,g) PG contents (scale bar: 1 cm). h) Circular dichroism spectra of HPC/PG mesophases with: different i) HPC and ii) PG contents.

HPC/PG mesophase materials were prepared by dissolving HPC (above a threshold concentration) in a mixed solution containing PG and deionized water. In this system, HPC molecules can self‐assemble spontaneously into a mesophase with a cholesteric helical arrangement (**Figure** **1**a). From the scanning electron microscopy (SEM) image, the periodic stacking structure of the cholesteric arrangement is confirmed (Figure 1b). The intense birefringence of the cholesteric phase can be detected from polarizing optical microscope (POM) images of HPC/PG mesophases (Figure 1c and Figure S1, Supporting Information). The results reveal the incorporation of PG does not inhibit the formation of the cholesteric structure of HPC, which is important for photonic properties.

Structural coloration of photonic materials is regulated through the selective reflection of visible light. The corresponding reflection wavelength (*λ*) can be estimated with the De Vries law as follows

(1) $$\lambda \;=\;n_{\mathrm{avg}}\;P\;\cos\theta \;$$

where *n*_avg_ represents the average refractive index of the cholesteric material; *P* is the helical pitch defined as the repeating distance of the cholesteric periodicity; *θ* refers to the angle between the reflection light and the cholesteric helix axis. At a fixed viewing angle, the corresponding *λ* of the cholesteric phase depends on the *n*_avg_ and *P*, which are relevant to many factors, including molecular weights, concentrations, and molecular interactions between multicomponents.^[^ ^13^, ^49^ ^]^ Therefore, different structural colors can be generated by the HPC/PG mesophases with different HPC and PG contents. The as‐prepared samples are named HPC*_x_*/PG*_y_* (*x*: the mass content of HPC in mesophase; *y*: the mass content of PG in total liquid solvent). As shown in Figure 1d, with increasing the HPC concentration from 58 to 68 wt%, the bright structural color of the mesophase changes from red to violet. As determined using UV‐vis extinction spectroscopy, the maximum wavelength (*λ*_max_) of sharp extinction peaks shifts from 710 to 396 nm as a function of the HPC content (Figure 1e and Figure S2, Supporting Information). Moreover, by controlling the PG content within 0–40 wt%, vivid colors of the HPC/PG mesophases are regulated from the violet to chartreuse colors (Figure 1f). The corresponding *λ*_max_ locates at 434, 451, 479, 506, and 539 nm, respectively, at the PG content of 0, 10, 20, 30, and 40 wt% (Figure 1g and Figure S3, Supporting Information). It should be noted that vivid colors are only observed with a right‐handed circular polarizer rather than a left‐handed circular polarizer (Figure S4, Supporting Information), indicating that the mesophases possess right‐handed chiral cholesteric architectures that can selectively reflect right‐handed circularly polarized light. Such chiral structures are verified by the circular dichroism (CD) analysis (Figure 1h), where strong and tunable positive signals are seen for the mesophases with different i) HPC and ii) PG contents.

**Figure 2:**
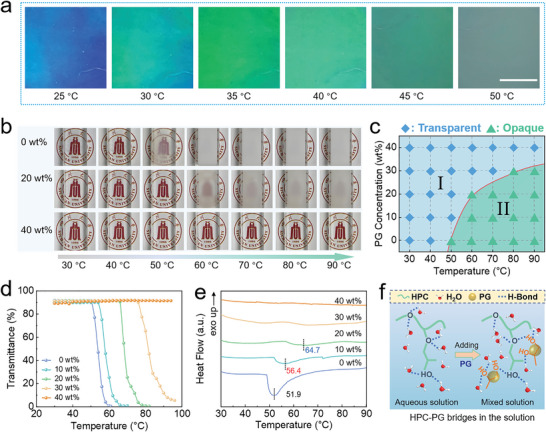
Phase transition behaviors. a) Structural colors of HPC_64_/PG_0_ mesophase at different temperatures. b) Photographs of HPC/PG solutions containing 0, 20, and 40 wt% PG at different temperatures. c) A phase diagram of HPC/PG solutions. I) The region with no phase separation; II) The region with phase separation behaviors. d) Turbidity at the wavelength of 600 nm and e) DSC curves of HPC/PG mesophases as a function of the temperature and PG content. f) A proposed mechanism to the increase in *T*_cp_ of HPC in the presence of PG.

In addition to the composition, environment temperatures can be also used to adjust the structural color of HPC mesophases, i.e., the red shift with an increase in the temperature; this can be attributed to the increase in the helix pitch as a result of changes in the HPC intermolecular force.^[^ ^13^, ^50^ ^]^ However, the structural color fades and turns to white once the temperature increases to 50 °C (**Figure** **2**a). This phenomenon can be explained by the lowest critical solution temperature (LCST) behavior of HPC.^[^ ^51^ ^]^ The temperature increase induces the molecular agglomeration and macroscopic phase transition, causing the destruction in the cholesteric structure and the failure of structural colorations. Therefore, the LCST determines the upper limit for temperature responses of the HPC photonic crystals.^[^ ^52^, ^53^ ^]^

Phase transitions are obviously seen from uniform transparent solutions to nonuniform opaque phases (Figure 2b,c and Figure S5, Supporting Information). The phase transition temperature of HPC solutions (0.5 wt%) is dependent on the PG contents, i.e., from 50 to 80 °C with the PG content increases from 0 to 30 wt%. Notably, in the case of 40 wt% PG content, no obvious phase transition occurs even at 90 °C; this is nearly the upmost temperature limit to detect phase transitions for aqueous polymer solutions.^[^ ^54^ ^]^ The phase transition behavior of the HPC/PG solutions was further evidenced optically with turbidimetry, from which the cloud point temperature (*T*_cp_, the temperature at which the transmittance reached 50%) can be determined. As shown in Figure 2d and Figure S6 in the Supporting Information, *T*_cp_ of HPC/PG solutions increases from 54 to 82 °C until unmeasurable at high PG contents. Additionally, obvious endothermic peaks are detected from differential scanning calorimeter (DSC) curves, and the peak area decreases with the increase in the PG content (Figure 2e); these results are in good agreement, suggesting that the phase transition behavior, especially the transition temperature, can be adjusted by controlling the PG content.

As reported before,^[^ ^55^, ^56^ ^]^ the control on *T*_cp_ is manipulated by the enthalpic (Δ*H*_m_) or entropic (Δ*S*_m_) during the phase transition via adjusting the competition between weak interactions, including hydrogen bonds (H‐bonds) and electrostatic interactions between polymer–solvent or/and polymer–polymer interactions. In the HPC/PG solution, H‐bonds between HPC and PG is stronger than those between HPC and water, thus HPC‐PG bridges are formed, improving the interaction between HPC and water molecules (Figure 2f). As a result, the more negative Δ*H*_m_ leads to an increase in *T*_cp_ and the upper limit temperature of the HPC mesophases.

**Figure 3:**
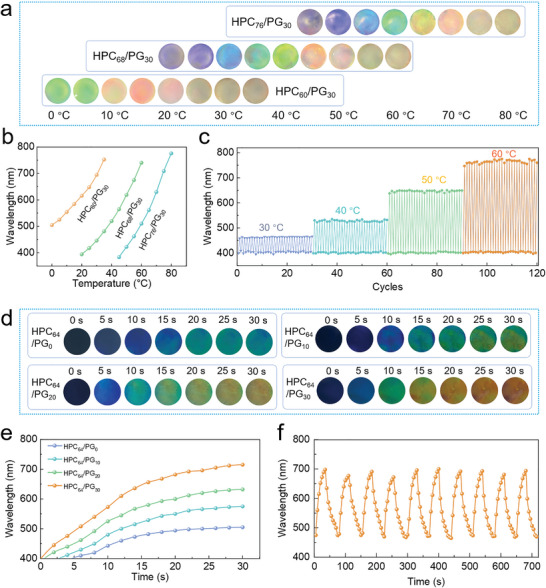
Thermally responsive performances. a) Optical images illustrating color variations of HPC/PG mesophases under different temperatures. b) The *λ*_max_ of HPC/PG mesophases as function of temperature. c) Cyclic color‐changing behaviors of HPC_68_/PG_30_ under different thermal‐stimuli. d) Optical images illustrating dynamic color variations of HPC_64_/PG mesophases with different PG contents upon heating from 5 to 45 °C. e) The corresponding *λ*_max_ of HPC_64_/PG mesophases as function of time. f) Response behavior of HPC_64_/PG_30_ under cyclic temperature alternations.

Temperature response behaviors of HPC/PG mesophases were further evaluated at different HPC and PG contents. Considering the no *T*_cp_ at 40 wt% PG, its content is set no higher than 30 wt%. As shown in **Figure** **3**a and Movie S1 in the Supporting Information, the structure color of HPC_60_/PG_30_ shifts from green to red with the increase in the temperature from 0 to 35 °C. Similar red‐shifting phenomena are also observed for HPC_68_/PG_30_ and HPC_76_/PG_30_; however, the response‐temperature ranges are different, i.e., 20–60 °C and 45 to 80 °C, respectively, and the color range is extended to purple‐to‐red. The corresponding *λ*_max_ of the HPC/PG mesophases are recorded in Figure 3b and Figure S7 in the Supporting Information. In accordance with the thermally induced color changes, *λ*_max_ of HPC_60_/PG_30_, HPC_68_/PG_30_, and HPC_76_/PG_30_ locates within 505–752, 395–740, and 384–776 nm, respectively. Moreover, the temperature responses are highly stable and reversible during cyclic temperature variations for at least 120 runs (Figure 3c and Figure S8, Supporting Information). It is worth noting that, as a result of the high and widely tunable *T*_cp_ for aqueous HPC mesophases, this work realizes an ultrawide temperature response range within the visual‐color region, as compared to previously reported photonic crystals (Figure S9 and Table S1, Supporting Information).

In addition to the upper limit temperature, more interestingly, the response rate can be adjusted by the PG contents. Since PG has a higher refractive index than water, the *n*_avg_ of HPC/PG mesophases increases with the PG content (Figure S10, Supporting Information). During the dynamic heating process to 45 °C, red‐shifted color changes are observed (Figure 3d). Within the same time period of 30 s, a higher PG content is favorable to a faster and more obvious color change. The quantitative analysis on the response rate is shown in Figure 3e, where the average response rates are calculated to be 3.7, 6.0, 8.1, and 10.6 nm s^−1^, respectively, at the PG contents of 0, 10, 20, and 30 wt%; such performances are superior over relevant works on photonic crystals (Figure S11 and Table S2, Supporting Information). Furthermore, during the cyclic heating–cooling process, reversible color changes are determined, and response rates are well maintained (Figure 3f).

**Figure 4:**
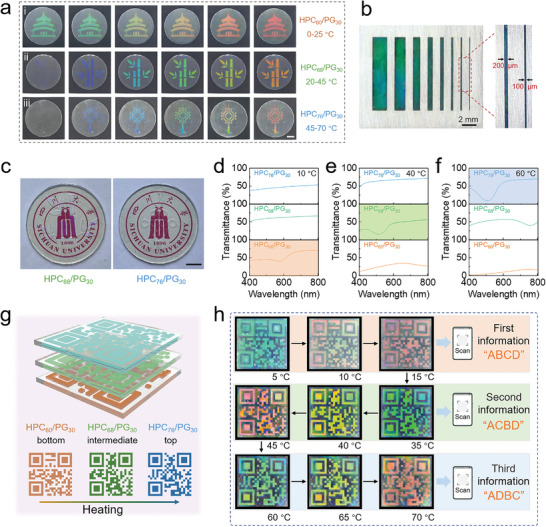
Temperature‐resolved multilevel information encryption/decryption. a) Photographs of i) the Temple of Heaven pattern formed with HPC_60_/PG_30_ at 0 to 30 °C; ii) bamboo pattern formed with HPC_68_/PG_30_ at 20 to 45 °C; iii) Chinese knot pattern formed with HPC_76_/PG_30_ at 40 to 70 °C. b) Resolution of HPC_64_/PG_30_ photonic ink. c) Transmittance and absorbance of HPC_68_/PG_30_ with different thicknesses. Transmission curves of HPC_60_/PG_30_, HPC_68_/PG_30_, and HPC_76_/PG_30_ mesophases at d) 10, e) 40, and f) 60 °C. g) Schematic illustration and h) practical application of multilevel information encryption and decryption at different temperatures. Scale bar: 1 cm.

Taking advantage of the structural color change and tunable temperature response, the HPC/PG mesophases can be directly used as a photonic ink for dynamic display and multilevel encryption/decryption. The ink was filled into a 3D‐printed transparent mold and sealed with polyethylene terephthalate (PET) films, forming pre‐setting patterns (Figure S12, Supporting Information). As shown in **Figure** **4**a, impressively, the Temple of Heaven, bamboo, and Chinese knot patterns depicted using HPC_60_/PG_30_, HPC_68_/PG_30_, and HPC_76_/PG_30_, respectively, present dynamic structural color switching observed to naked eyes at different temperature ranges. The resolution of the patterning/painting was determined, in terms of the line width, to be 100 µm (Figure 4b); this is sufficient for general applications. Patterns can be also created on the cardboard, PET, and polymethyl methacrylate films using the 3D printer on the basis of the fluidity and viscosity of the ink (Figure S13, Supporting Information). Additionally, the HPC/PG inks can be used for constructing QR codes (Figure S14, Supporting Information), which are displayed or hidden through temperature controls, i.e., temperature‐dependent information decryption. In addition, when using the inks to depict patterns, the patterns only appear in the corresponding temperature range, and there is no crosstalk among different colors (Figure S15, Supporting Information); the performance provides an opportunity for selectively storing different pieces of information into multiple channels and avoids confusion or misunderstanding for information encryption.

Prior to the further investigation on using the inks for multilevel information encryption/decryption, transparency of patterns depicted with the inks should be analyzed. As shown in Figure 4c and Figure S16 in the Supporting Information, pictures underneath the inks are seen clearly, especially at the ink thickness below 2 mm. More importantly, the transmittance ensures that the color of the response layer is not shaded (Figure 4d–f and Figure S17, Supporting Information), namely, only a single channel can be recognized at 10, 40, and 60 °C while two channels appear simultaneously colors at 20, 30, and 50 °C that are not desired for information access. The unique features enable multilevel information storages at a stacking mode. As shown Figure 4g, a triple‐layer label is assembled with three HPC/PG QR codes. These QR codes can store the “ABCD,” “ACBD,” and “ADBC” information independently, which were set, respectively, at bottom, middle, and top layers. As shown in Figure 4h and Movie S2 in the Supporting Information, 1) at the temperature between 5 and 15 °C, the top and middle QR codes are transparent and colorless, while only the bottom QR code at the bottom presents visible patterns that can read mobile devices to give the “ABCD”; 2) within 35 to 45 °C, the middle QR code shows visible colors while the top one keeps transparent, so the corresponding hidden “ACBD” information can be read out; 3) within 60 to 70 °C, the visible color of the top QR code appears, enabling the identification of the “ADBC” information. It should be noted that, at temperatures outside these ranges, multiple QR codes appear simultaneously, and the overlapping results in the failure to read the information (Figure S18, Supporting Information). Such encryption approach can hide correct information in multiple pieces of information and can also consolidate multiple messages with a single “multilayer”‐code; only at the definite temperature point and following correct heating procedures, true encrypted information can be read out.

**Figure 5:**
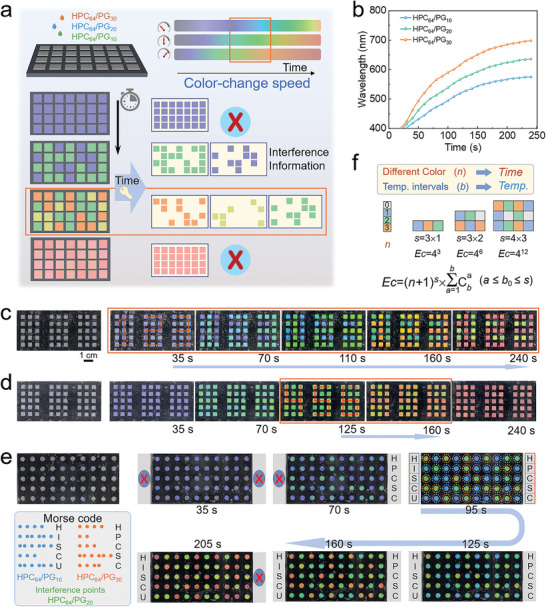
Time‐resolved information encryption/decryption. a) Design of encryption labels. b) Dynamic temperature responses of HPC/PG mesophases with different PG contents. Dynamic decryption processes of c) “123”‐information using HPC_64_/PG_10_ and HPC_64_/PG_30_ as codes and d) “456”‐information using HPC_64_/PG_20_ and HPC_64_/PG_30_ as codes. e) Decryption of Morse codes constructed using HPC_64_/PG_10_, HPC_64_/PG_20_, and HPC_64_/PG_20_. f) Encoding capability of HPC/PG mesophases. Scale bar: 1 cm.

In addition to the temperature resolution, the information encryption and decryption can be also realized by the control on time points (**Figure** **5**a). Here, we consider to use the mesophases with different PG contents, because of similar initial colors but obviously different color‐changing rates (Figure 5b). When the security information is stored with HPC_64_/PG_30_ and the interference information with HPC_64_/PG_10_, no clear information can be distinguished until decoding for 35 s (Figure 5c and Movie S3 in the Supporting Information). Furthermore, if adding HPC_64_/PG_20_ (small speed difference with HPC_64_/PG_30_) as the second interference phase, the encrypted information, “123,” can be only read out in a certain time period (125 to 160 s), as shown in Figure 5d and Movie S4 in the Supporting Information; however, at the time point outside the range, the three phases present almost indistinguishable purple/blue or pink/red colors so that the information is hidden and cannot be acquired. The encryption/decryption can be reversibly manipulated at least 20 cycles (Figure S19, Supporting Information). Interestingly, the HPC/PG mesophases can be further used for more complex encryption, i.e., Morse codes. According to the code rules, two pieces of information (“HISCU” and “HPCSC”) were loaded with HPC_64_/PG_10_ and HPC_64_/PG_30_, respectively, in the form of pixel points, and HPC_64_/PG_20_ was input as the interference phase. The pixel label containing the three mesophases is “colorless” initially; after treating at 45 °C for 70 s, different colors of the mesophases appear, enabling the information decryption; however, the decryption is achieved within 110 s (from 95 to 205 s), because after 205 s, the colors of HPC_64_/PG_20_ and HPC_64_/PG_30_ cannot be distinguished, making the information hidden again (Figure 5e and Movie S5 in the Supporting Information).

The HPC/PG mesophases with the temperature‐resolved and time‐resolved encryption will greatly increase the complexity of decryption. Accurate decryption details, including the appropriate temperature and time points, are required to decrypt hidden messages. If a higher number (*n*) of different colors within the visible light range (adjusted by different color‐changing rates as a result of controlling the PG content) that can be distinguished is achieved, more time‐dependent codes can be designed; if a larger number (*b*) of color‐transition‐temperature intervals (tuned by the HPC concentration) is realized, more temperature procedures acting as keys can be designed. Therefore, on the basis of temperature‐ and time‐dependent color control and advanced encryption design, more secure encryption/decryption with complex information‐storage codes and decryption pathways are expected to be enabled (Figure 5f). Moreover, the photonic inks have high long‐term stability under the storage in a sealed bottle; however, if the inks are placed at ambient condition without sealing, an obvious blue shift is observed within 8 h due to the water loss (Figure S20, Supporting Information). As we know, the information encryption technology requires anticounterfeiting materials with high security, large storage capacity, convenient reading, high stability, and low cost. As a promising photonic material, HPC has advantages of renewability, biodegradability, nontoxicity, and easy processability, which offer its great potential as photonic inks for information encryption and anticounterfeiting.

## 3 Conclusion

In conclusion, we demonstrate a new photonic anticounterfeiting ink with dynamic programming of colors and temperature‐/time‐resolved encryption. The structural color and transparency of the ink can be modulated by controlling the concentrations of HPC and PG as well as the ambient temperature. The interaction between HPC and PG enables color variations within a wide temperature range, and the temperature response range and rate are regulated by the HPC and PG phases, respectively. These features enable the encryption of multiple information with the photonic inks into different channels, where single or multiple messages can be recognized simultaneously. Such merit realizes distinguishing “true” information from complex multiple “false” information at appointed temperature and time points. The findings we believe offer a new starting point for photonic materials for synergistically temperature‐ and time‐resolved information coding/encoding, bringing inspirations to high‐end anticounterfeiting technologies.

## 4 Experimental Section

### Materials

Hydroxypropyl cellulose (HPC), of which the molecular weight and substitution degree of hydroxypropyl were determined by gel permeation chromatography (GPC) and ^1^H NMR and to be 199 000 and 1.68, was purchased from Tokyo Chemical Industry Co., Ltd. 1,2‐propanediol (PG) was purchased from Kelong Chemical Co., Ltd. 3D printing resin was purchased from Anycubic Technology Co., Ltd. Aerosol paint (black, NO. 39) was purchased from Sanhe Chemical Technology Co., Ltd. All materials were used as received without any further purification. Deionized water used in all experiments was collected from a Milli‐Q Plus water purification system (Millipore, USA).

### Preparation of HPC/PG Mesophases

HPC/PG mesophases were prepared by mixing HPC and PG with deionized water at different weight ratios of HPC and PG (Table S3, Supporting Information). After stirring for 2 h, the samples were degassed by centrifugation at 5000 rpm and then placed at 4 °C for 4 weeks until coloration.

### Fabrication of Patterns, QR Codes, and Multipixel Displays

Molds of different patterns, QR codes, and multipixel plates with controlled sizes were made from a clear resin using a 3D printer (Photon Mono X 6K, Anycubic). Using a micrometer, the thickness and inner chamber height of the modes were determined to be 1.484 ± 0.003 and 1.115 ± 0.035 mm, respectively. The chambers of the molds were filled with the HPC/PG mesophases and then encapsulated with PET films (for instance, the preparation of multipixel plates is shown in Figure S21, Supporting Information).

### Characterization

Microstructures were determined on a scanning electron microscope (JSM‐5900LV, Japan). UV‐vis extinction and transmittance spectra were collected with an UV‐vis spectrophotometer (Varian Cary 50, USA) equipped with a programmable temperature controller (PolyScience, USA) from 0 to 100 °C. POM was conducted with a polarizing optical microscope (ZEISS Axio Scope.A1, Germany). CD spectra were collected on a J‐1500 CD spectrometer (Jasco, Tokyo, Japan). Reflectance spectra were measured using a high‐sensitivity spectrometer (Ocean Optics Maya‐2000 pro, USA) with an angle‐resolved instrument (Wyoptics RS, China). The phase transition temperature was measured using a DSC (Q2000, TA Instruments) from 30 to 100 °C at a heating rate of 10 °C min^−1^. Refractive indexes were observed using a WAY‐2 W Abbe refractometer. IR thermal images were taken with a forward‐looking IR camera (FLIR T420, USA). Photographs and videos were taken with a digital camera and a black background (Apple iPhone 12).

## Conflict of Interest

The authors declare no conflict of interest.

## Supporting information

Supporting Information

Supplemental Movie 1

Supplemental Movie 2

Supplemental Movie 3

Supplemental Movie 4

Supplemental Movie 5

## Data Availability

The data that support the findings of this study are available from the corresponding author upon reasonable request.
