# Supplementary material for: Sophisticated yet Convenient Information Encryption/Decryption Based on Synergistically Time‐/Temperature‐Resolved Photonic Inks
Source: Adv Sci (Weinh). 2022 Dec 11;10(5):2206290. doi: 10.1002/advs.202206290 (PMC9929127; doi:10.1002/advs.202206290)
Supplement: Supplementary file 1 — Supporting Information [file ADVS-10-2206290-s003.pdf]

ADVANCED SCIENCE
Open Access

Supporting Information

for *Adv. Sci.*, DOI 10.1002/advs.202206290

Sophisticated yet Convenient Information Encryption/Decryption Based on Synergistically Time-/Temperature-Resolved Photonic Inks

*Dong Li, Jia-Min Wu, Zheng-Hong Liang, Lin-Yue Li, Xiu Dong, Si-Kai Chen, Teng Fu, Xiu-Li Wang, Yu-Zhong Wang and Fei Song**

Supporting Information

Sophisticated yet Convenient Information Encryption/Decryption Based on Synergistically Time-/Temperature-Resolved Photonic Inks

*Dong Li, Jia-Min Wu, Zheng-Hong Liang, Lin-Yue Li, Xiu Dong, Si-Kai Chen, Teng Fu, Xiu-Li Wang, Yu-Zhong Wang, and Fei Song**

D. Li, J. Wu, Z. Liang, L. Li, X. Dong, Dr. S. Chen, Dr. T. Fu, Prof. X. Wang, Prof. Y. Wang, Prof. F. Song
The Collaborative Innovation Center for Eco-Friendly and Fire-Safety Polymeric Materials (MoE), National Engineering Laboratory of Eco-Friendly Polymeric Materials (Sichuan), State Key Laboratory of Polymer Materials Engineering College of Chemistry, Sichuan University, Chengdu 610064, China

*E-mail: songfei520@gmail.com

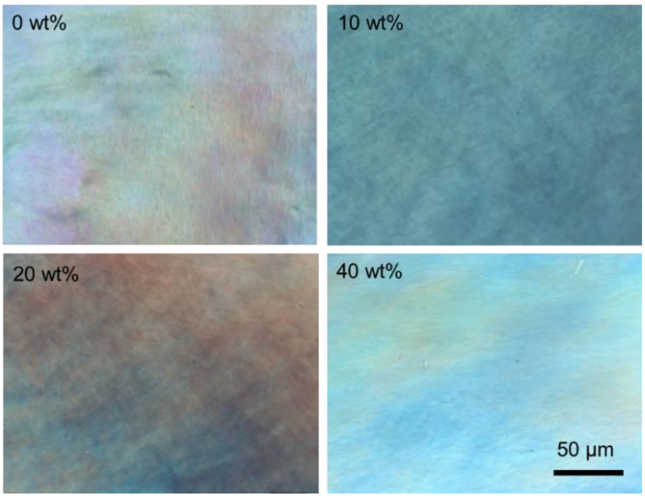

Figure S1. POM images of HPC/PG mesophases with different PG contents.

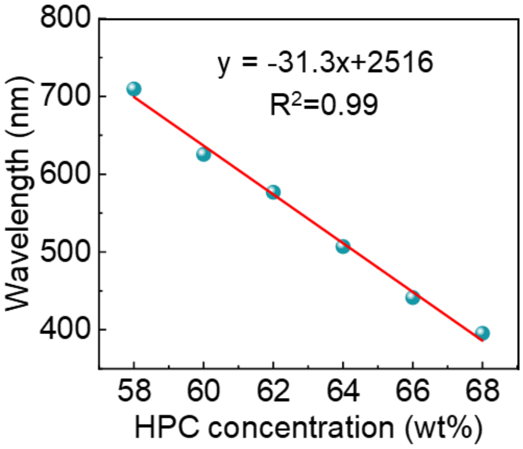

Figure S2. λ_{max} of HPC/PG mesophases as function of the HPC concentration.

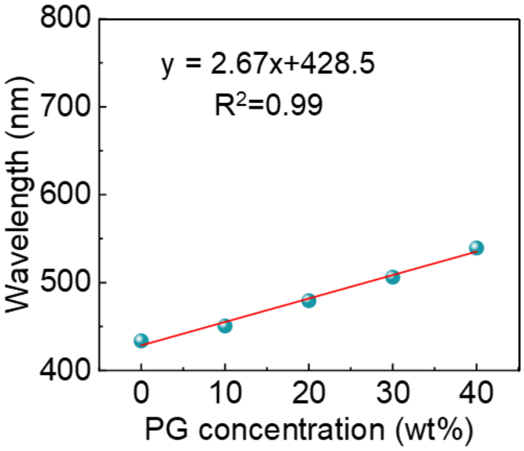

Figure S3. λ_{max} of HPC/PG mesophases as function of the PG concentration.

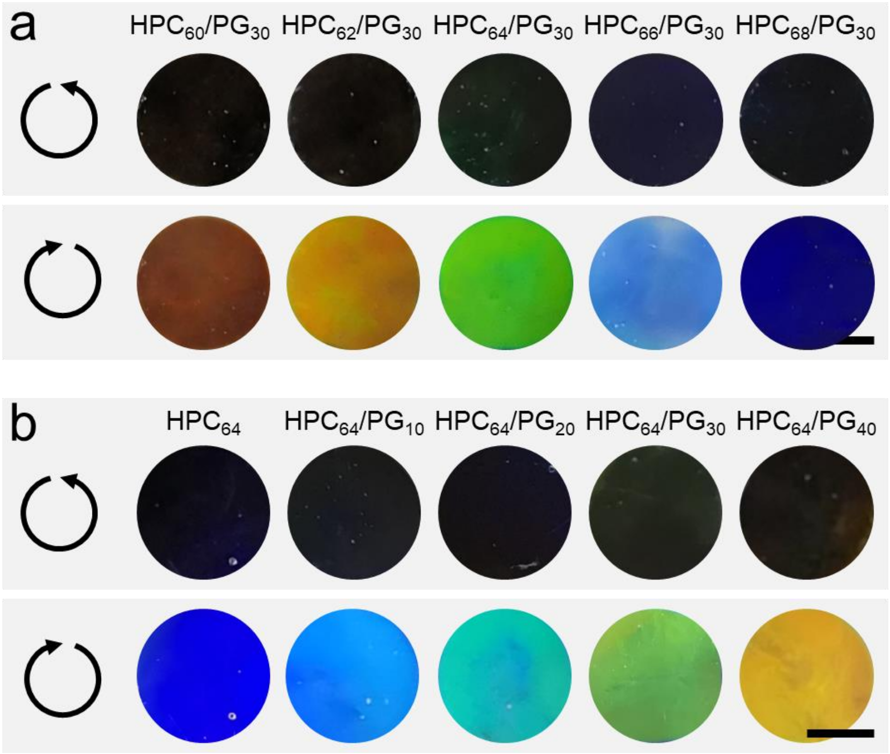

Figure S4. Photographs of HPC/PG mesophases with different (a) HPC and (b) PG contents viewed under left-handed and right-handed circular polarized filters. Scale bar: 1 cm.

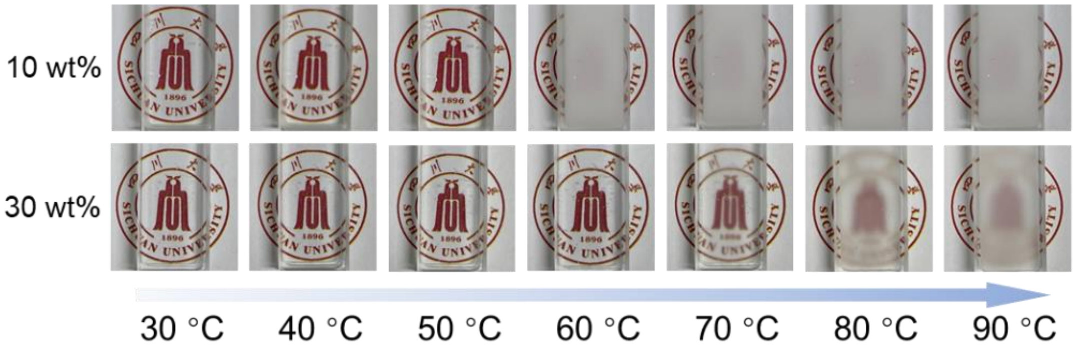

Figure S5. Photographs of the HPC/PG solution containing 10 wt% and 30 wt% PG at different temperatures.

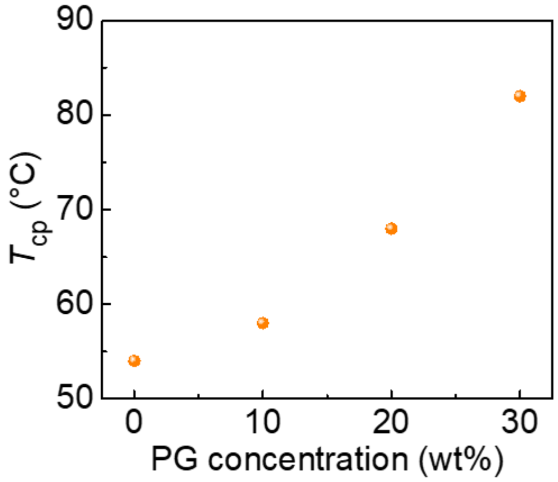

Figure S6. T_{cp} of 0.5 wt% HCP in a mixed solution with different PG contents.

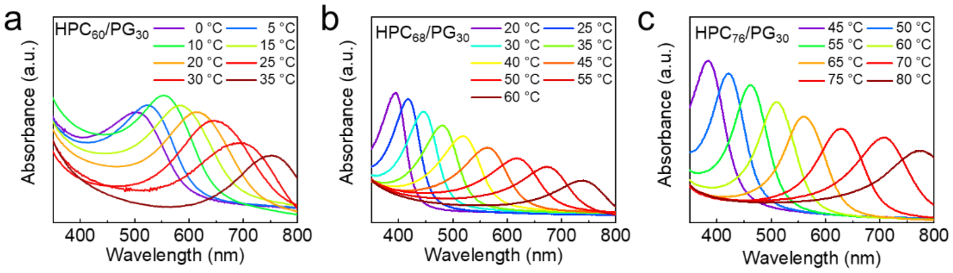

Figure S7. UV−vis extinction spectra of HPC_{60}/PG_{30}, HPC_{68}/PG_{30}, and HPC_{76}/PG_{30} at different temperatures.

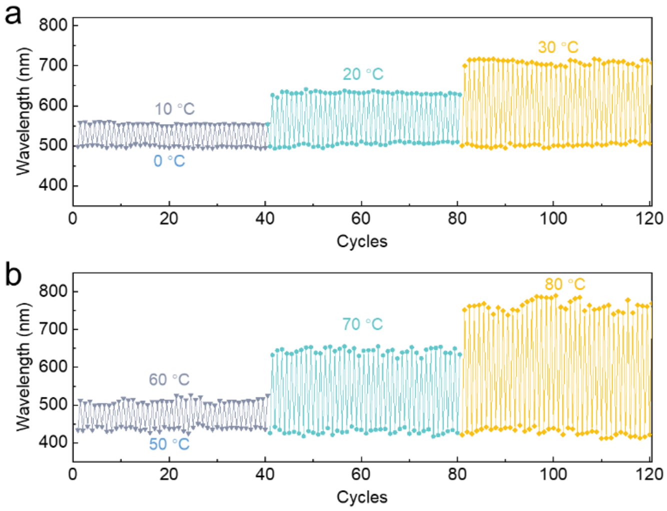

Figure S8. The λ_{max} of (a) HPC_{60}/PG_{30} and (b) HPC_{76}/PG_{30} during different cyclic heating-cooling runs.

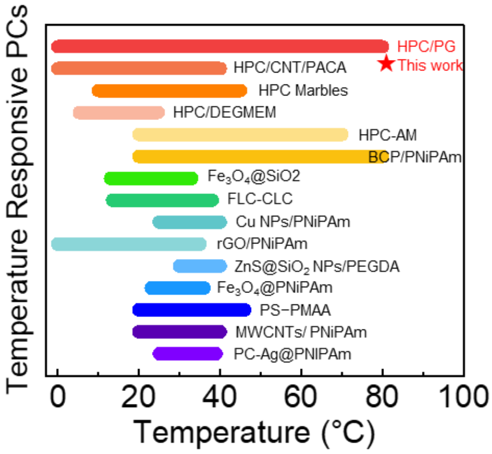

Figure S9. Comparison of temperature response ranges: HPC/PG mesophases *vs* other photonic crystals.

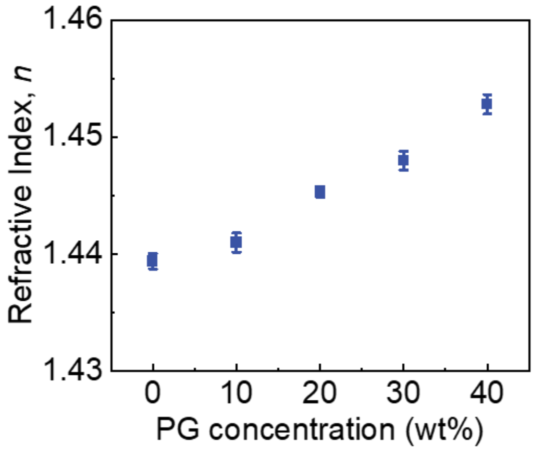

Figure S10. Average refractive indexes (n_{avg}) of HPC/PG mesophases with different PG contents.

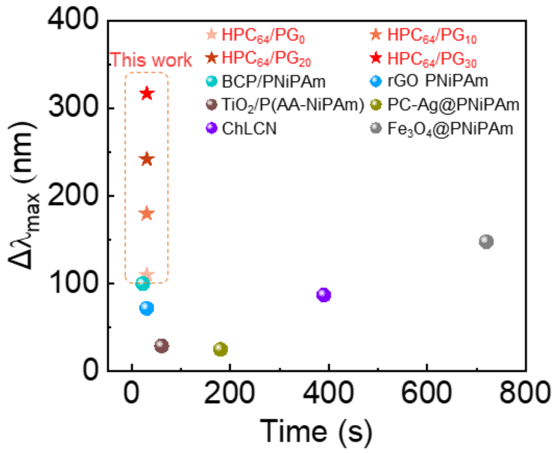

Figure S11. Comparison of the response time and wavelength variation: HPC/PG mesophases *vs* other photonic crystals.

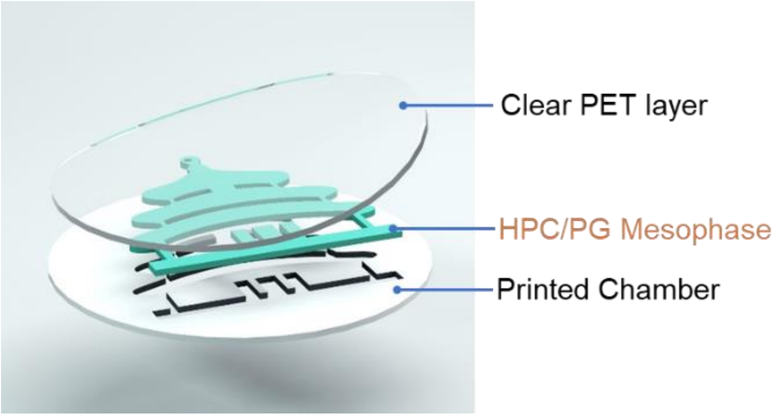

Figure S12. Architectures of designed patterns.

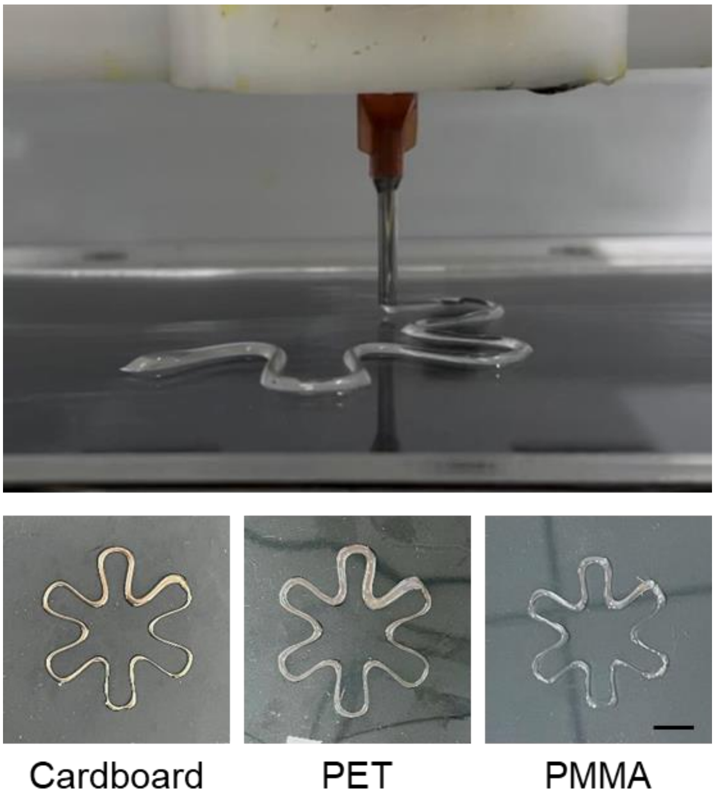

Figure S13. 3D printing of the HPC/PG inks on cardboard, PET and PMMA films. Scale bar is 1 cm.

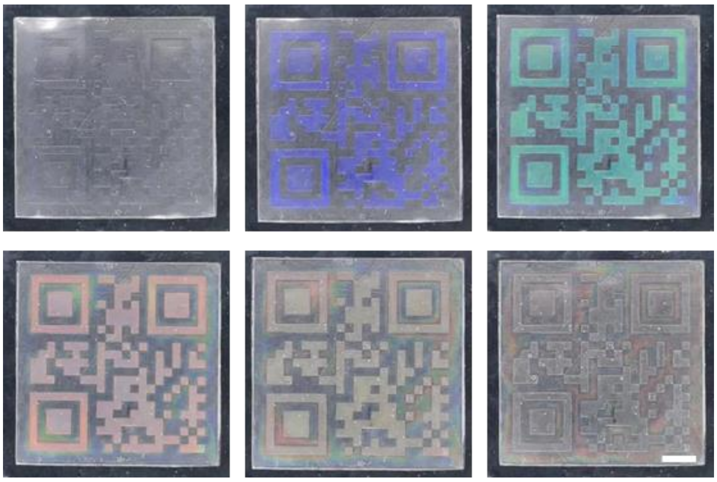

Figure S14. Color switching of a QR code upon heating. Scale bar: 1 cm.

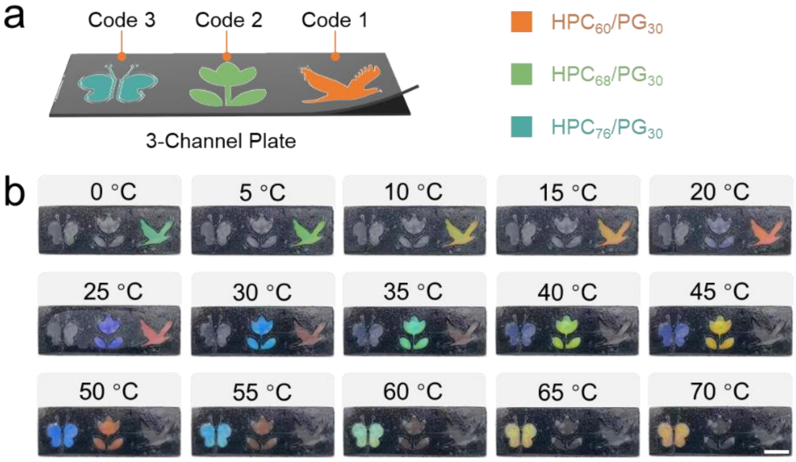

Figure S15. (a) Schematic illustration of a 3-channel label. (b) Changes in the patterns of the label at different temperatures. Scale bar: 1 cm.

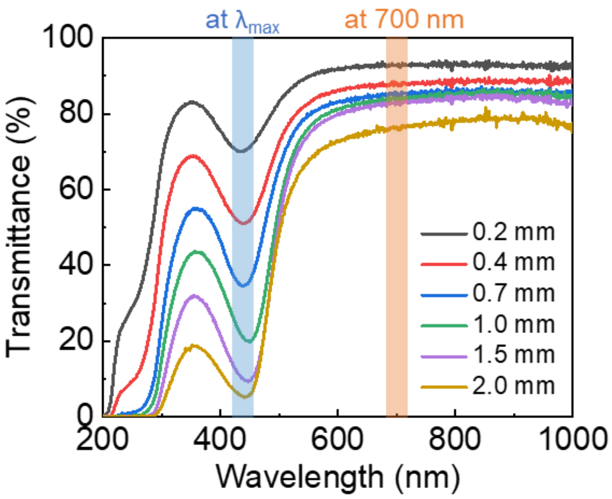

Figure S16. Transmittance curves of the HPC_{68}/PG_{30} mesophase in different-thickness cells.

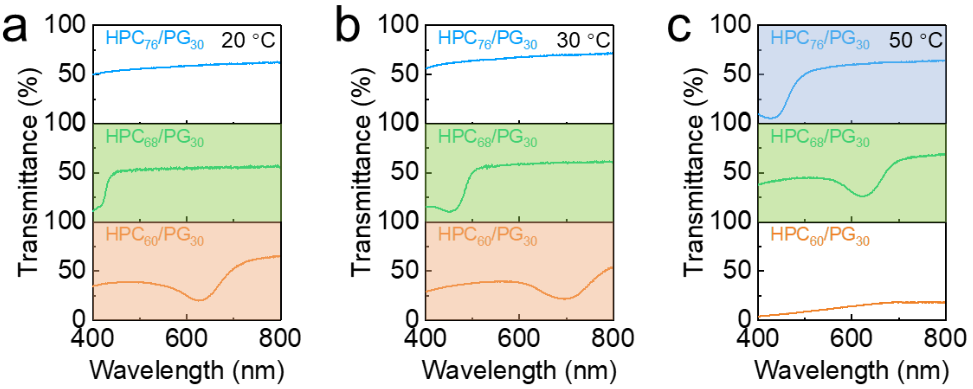

Figure S17. Transmittance curves of HPC_{60}/PG_{30}, HPC_{68}/PG_{30}, and HPC_{76}/PG_{30} mesophases at (a) 20 °C, (b) 30 °C, and (c) 50 °C.

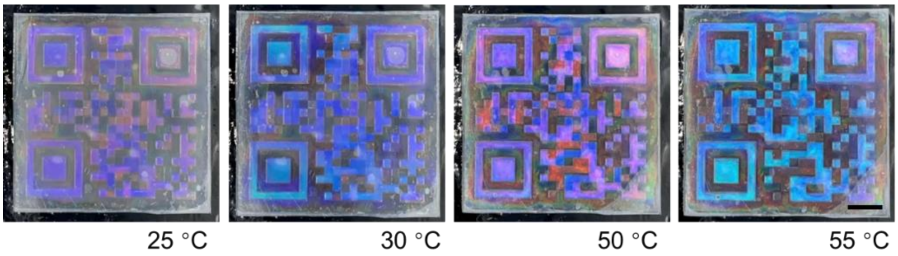

Figure S18. Photographs of a triple-layer label at different temperatures. Scale bar: 1 cm.

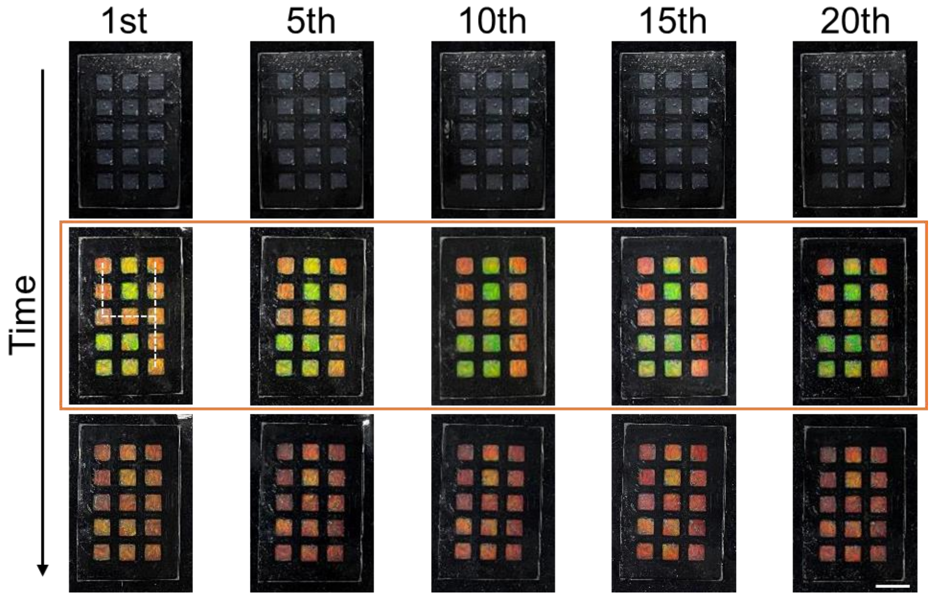

Figure S19. Stability tests for the encryption information by repeatedly heating decryption. Scale bar is 1 cm.

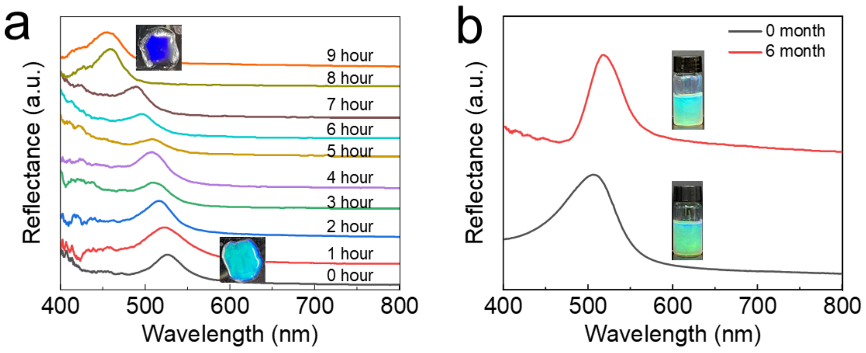

Figure S20. Long-term stability of the HPC/PG ink (a) without or (b) with sealing.

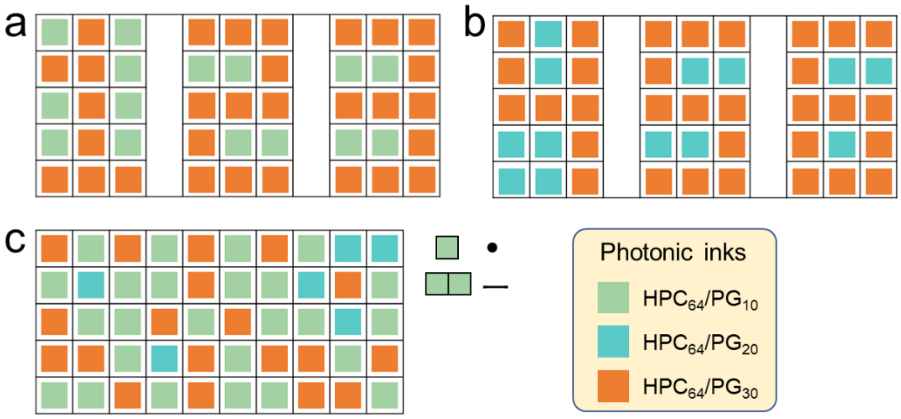

Figure S21. Schematic illustration of encryption labels of (a) "123"-information, (b) "456"-information, and (c) Morse codes constructed with different HPC/PG mesophases.

Table S1. Comparison of temperature response behaviors of this work with relevant reports

Materials	Response range (°C)	$\Delta\lambda_{max}$ (nm)	Ref.
HPC/PG mesophase	0-80	400	**This work**
HPC/CNT/P(AAm-co-AAc)	0-40	260	**1**
HPC liquid marbles	10-45	205	**2**
HPC/DEGMEM mesophase	5.3-24.7	230	**3**
HPC-AM mesophase	20-70	400	4
BCP/PNiPAm	20-80	100	5
Fe_3O_4@SiO_2 colloids	13-33	124	6
FLC-CLC	13.5-38	400	7
Cu NPs/PNiPAm	25-40	180	8
rGO/PNiPAm	0-35	195	9
ZnS@SiO2 NPs/PEGDA	30-40	100	10
Fe3O4@ PNiPAm	23-36	148	11
PS−PMAA	20-46	91	12
MWCNTs/ PNiPAm	20-40	300	13
PC-Ag@PNIPAm	25-39	25	14

Table S2. Comparison of temperature response time and wavelength variation of this work with relevant works

Materials	Response time (s)	$\Delta\lambda_{max}$ (nm)	Ref.
HPC_{64}/PG_0	30	110	**This work**
HPC_{64}/PG_{10}	30	180	**This work**
HPC_{64}/PG_{20}	30	242	**This work**
HPC_{64}/PG_{30}	30	317	**This work**
BCP/PNiPAm	22	100	5
rGO/PNiPAm	30	72	15
TiO_2/P(AA-NiPAm)	60	29	16
PC-Ag@PNiPAm	180	25	14
ChLCN	390	87	17
Fe_3O_4@PNiPAm	720	148	11

Table S3. Components of photonic inks

Sample	HPC_{60}/ PG_{30}	HPC_{62}/ PG_{30}	HPC_{64}/ PG_{30}	HPC_{66}/ PG_{30}	HPC_{68}/ PG_{30}	HPC_{64}/ PG_{0}	HPC_{64}/ PG_{10}	HPC_{64}/ PG_{20}	HPC_{64}/ PG_{40}
m_{HPC}/g	3.00	3.10	3.20	3.30	3.40	3.20	3.20	3.20	3.20
m_{water}/g	1.40	1.33	1.26	1.19	1.12	1.80	1.62	1.44	1.08
m_{PG}/g	0.60	0.57	0.54	0.51	0.48	0.00	0.18	0.36	0.72

Movie 1.

Color variations of the HPC/PG mesophases upon heating.

Movie 2.

Temperature-resolved multilevel information encryption and decryption.

Movie 3.

Time-resolved decryption of “123”-information.

Movie 4.

Time-resolved decryption of “456”-information.

Movie 5.

Time-resolved decryption of Morse codes.

References

[1] Z. Zhang, Z. Chen, Y. Wang, Y. Zhao, *Proc Natl Acad Sci U S A* **2020**, 117, 18310.
[2] M. Anyfantakis, V. S. R. Jampani, R. Kizhakidathazhath, B. P. Binks, J. P. F. Lagerwall, *Angew. Chem. Int. Ed.* **2020**, 59, 19260.
[3] R. Chiba, Y. Nishio, Y. Sato, M. Ohtaki, Y. Miyashita, *Biomacromolecules* **2006**, 7, 3076.
[4] Z. Zhang, Z. Chen, Y. Wang, Y. Zhao, L. Shang, *Adv. Funct. Mater.* **2021**, 31, 2107242.
[5] T. H. Park, H. Eoh, Y. Jung, G. W. Lee, C. E. Lee, H. S. Kang, J. Lee, K. B. Kim, D. Y. Ryu, S. Yu, C. Park, *Adv. Funct. Mater.* **2020**, 31, 2008548.
[6] W. T. Wang, X. Q. Fan, F. H. Li, J. J. Qiu, M. M. Umair, W. C. Ren, B. Z. Ju, S. F. Zhang, B. T. Tang, *Adv. Opt. Mater.* **2018**, 6, 1701093.
[7] S. A. Jiang, J. L. Chang, J. W. Lin, Y. S. Zhang, T. S. Mo, J. D. Lin, C. R. Lee, *Adv. Opt. Mater.* **2021**, 9, 2001796.
[8] M. Xiong, Y. Sheng, Y. Di, F. Xing, L. Yu, J. Zhang, W. Zhou, C. Liu, L. Dong, Z. Gan, *ACS Appl. Mater. Interfaces* **2021**, 13, 33566.
[9] L. Cai, Y. Wang, L. Sun, J. Guo, Y. Zhao, *Adv. Opt. Mater.* **2021**, 9, 2100831.
[10] Y. Wu, Y. Wang, S. Zhang, S. Wu, *ACS Nano* **2021**, 15, 15720.
[11] S. Shang, P. Zhu, H. Wang, Y. Li, S. Yang, *ACS Appl. Mater. Interfaces* **2020**, 12, 50844.
[12] H. Wang, S. Yang, S. N. Yin, L. Chen, S. Chen, *ACS Appl. Mater. Interfaces* **2015**, 7, 8827.
[13] Y. Xia, S. Gao, R. Yu, Z. Zeng, H. He, X. Zhou, Y. Hu, M. Cao, S. Wang, *ACS Appl. Polym. Mater.* **2021**, 3, 757.
[14] X. Fei, T. Lu, J. Ma, S. Zhu, D. Zhang, *Nanoscale* **2017**, 9, 12969.
[15] Y. Wang, J. Guo, L. Sun, H. Chen, Y. Zhao, *Chem. Eng. J.* **2021**, 415, 128978.
[16] C. Liu, C. Yao, Y. Zhu, J. Ren, L. Ge, *Sensors and Actuators B: Chemical* **2015**, 220, 227.
[17] O. M. Wani, A. P. H. J. Schenning, A. Priimagi, *J. Mater. Chem. C* **2020**, 8, 10191.
